# Renal “hyperfiltrators” are at elevated risk of death and chronic diseases

**DOI:** 10.1186/1471-2369-15-160

**Published:** 2014-10-02

**Authors:** Servet Altay, Altan Onat, Fatma Özpamuk-Karadeniz, Yusuf Karadeniz, Tuğba Kemaloğlu-Öz, Günay Can

**Affiliations:** Cardiology, Siyami Ersek Center for Cardiovascular Surgery, Istanbul, Turkey; Cardiology, Cerrahpaşa Medical Faculty, Istanbul University, Istanbul, Turkey; Public Health, Cerrahpaşa Medical Faculty, Istanbul University, Istanbul, Turkey; Department of İnternal Medicine, Haseki Training and Research Hospital, Istanbul, Turkey

**Keywords:** Autoimmune activation, Cardiovascular disease, Chronic obstructive pulmonary disease, Glomerular filtration rate, Heart failure, Renal hyperfiltration

## Abstract

**Background:**

The definition of glomerular hyperfiltration has not been agreed upon and the pathophysiological mechanisms have not been well explored. Low serum creatinine concentrations may be associated with increased risk of coronary heart disease (CHD) or cardiopulmonary events the impact of which needs further study.

**Methods:**

Consecutive applicants to a cardiovascular hospital free of moderate/severe chronic kidney disease (age 55.6 ± 8.2 years) were grouped into those without (“healthy”, n = 469) and with CHD (320 stable and acute coronary syndrome cases) at baseline and into sex-specific quartiles of CKD-EPI equation-estimated glomerular filtration rate (eGFR). New or recurrent cardiovascular (myocardial infarction, stroke, heart failure [HF]) events, obstructive pulmonary disease (COPD) and death were determined during 3-years’ follow-up.

**Results:**

Among 25 deaths and 75 cardiopulmonary events, HF was the leading nonfatal event. Age, serum uric acid and left ventricular ejection fraction proved the best independent inverse covariates of eGFR in the “healthy” sample. The highest eGFR quartile (“hyperfiltrators”), exhibiting significantly lower serum LDL-cholesterol levels, significantly predicted the combined outcome (at a RR of 6) in “healthy” subjects, after adjustment for sex, age, body mass index, smoking status and presence of hypertension. This finding was paralleled by the highest eGFR quartile calculated also by the MDRD equation, replicating this also in the CHD group.

**Conclusion:**

Renal “hyperfiltrators” represent individuals with autoimmune activation (involving serum creatinine, partly escaping assay), are misclassified into optimal renal function and actually are at significantly higher risk of death, HF or cardiopulmonary events. Low serum creatinine levels may represent a clue to the existence of autoimmune activation.

## Background

Renal hyperfiltration has been described to occur in renal and non-renal clinical conditions [1, 2]. The definition of glomerular hyperfiltration has not been agreed upon and the pathophysiological mechanisms have not been well explored [1], yet a glomerular filtration rate (GFR) 125 to 140 ml/min per 1.73 m^2^, or >2 SD above mean GFR in healthy individuals is a commonly adopted definition [2, 3]. In humans, renal hyperfiltration observed in early diabetes mellitus is a risk factor for the development of diabetic nephropathy [4]. More recently, renal hyperfiltration has been reported in obesity and the metabolic syndrome (MetS) [5–8]. In subjects with impaired fasting glucose, hyperfiltration occurred before the diagnosis of type-2 diabetes [7]. A “physiological” state of glomerular hyperfiltration occurs during pregnancy and after consumption of high-protein meals [1].

The pathogenesis of hyperfiltration is incompletely understood and attributed in diabetes to glomerular afferent and efferent arteriolar tone as well as to tubular factors; others have proposed a tubuloglomerular feedback hypothesis (reviewed in Ref. 2).

Information on the clinical significance and pathophysiology of glomerular hyperfiltration was extended by evidence reported in middle-aged Turkish adults [9] that low (as well as high) levels of creatinine (or “hyperfiltration”) were independently associated with future coronary heart disease (CHD) risk in women, as distinct from men. They ascribed the low measurements to partial inability to assay circulating creatinine, secondary to involvement in autoimmune activation. The group of the Turkish Adult Risk Factor (TARF) study later demonstrated that CHD likelihood in women in the lowest creatinine quartile was associated with CHD risk even in non-diabetic subjects without MetS [10], indicating that the underlying pathologic process had its onset already in “apparently healthy” female population at large.

Serum creatinine was not the only polypeptide/protein that was presumably comprised in autoimmune activation. Evidence for the involvement of other proteins such as lipoprotein[Lp] (a) [11] was available in the TARF cohort or of cystatin C and glycated hemoglobin in studies from other populations, upon which a unifying hypothesis of autoimmune activation underlying diverse chronic diseases was put forward by Onat and Can [12], further explained in the Discussion.

We aimed in this study to test in a community-based sample undergoing health checks at a large tertiary cardiovascular center whether subjects with low serum creatinine (or elevated eGFR, “hyperfiltrators”): a) had normal or increased risk for mortality and/or cardiovascular and chronic pulmonary events at an intermediate follow-up period, and b) which clinical and laboratory risk profile characterized them at baseline. These we analyzed after stratifying the study sample into quartiles of eGFR and the presence or absence of CHD which partly included cases of acute coronary syndrome (ACS). For estimating GFR, we have evaluated both the Modification of Diet in Renal Disease (MDRD) [13] and the Chronic Kidney Disease Epidemiology Collaboration (CKD-EPI) [14] equations.

## Methods

### Study sample

The baseline study sample is formed by consecutive outpatients and patients admitted in the first quarter of year 2010 to the S. Ersek Center for Cardiovascular Surgery who had no missing values for serum creatinine concentrations. This sample was drawn from a total of 1900 patients applying in the stated period. For reasons of lower risk of cardiopulmonary events or confounding problems of potential comorbidity, subjects aged <40, or >72 years and those with greater than mild renal dysfunction (<60 ml/min per 1.73 m^2^) (n = 207), decompensated heart failure (n = 120) and uncontrolled diabetes (HbA1c >10%; n = 92) were excluded, as were subjects with missing creatinine values (n = 494). This left 789 individuals to form the study sample. Follow-up consisted of up to 3.0 years. The study was approved by the Institutional Ethics Committee of the Dr. Siyami Ersek Training Research Hospital Center. Data recorded in the hospital charts were obtained.

### Consent statement

As this study is a retrospective study, informed consent was not obtained from the patients.

### Measurement of risk factors

Blood pressure (BP) was measured with an aneroid sphygmomanometer (Erka, Germany) in the sitting position on the right arm, and the mean of at least two recordings 5 min apart was registered. Body mass index (BMI) was computed from values of weight/height squared. Never, former and current smokers formed the categories of cigarette smoking.

*Blood samples*, collected after an overnight fast, were spun at 1000 g, and analyzed in a central laboratory. All laboratory determinations were performed using Siemens Healthcare Diagnostic Products kits and calibrators (Marburg, Germany). Serum creatinine concentrations were measured with the Jaffe method having a lower detection limit of 0.1 mg/dl and uric acid with the uricase method. Serum concentrations of total cholesterol, triglycerides and glucose were measured enzymatically. LDL- and high-density lipoprotein (HDL)-cholesterol were quantified directly with the elimination catalase method and Advia autoanalyzer. Concentrations of HbA1c were measured in whole blood agglutination inhibition and serum CRP with Latex-enhanced immunoturbidimetry.

Sixty-four percent of the study participants underwent two-dimensional and Doppler echocardiography by an experienced specialist using a Vivid 3 (GE Healthcare Systems, Piscataway, New Jersey, USA) device. Left ventricular ejection fraction (LVEF) was calculated using the modified Simpson’s method.

### Definitions

GFR (in ml/min per 1.73 m^2^) was estimated using a) the equation proposed by CKD-EPI for white females and males [14] which uses truncation for low creatinine values, and b) the abbreviated equation developed by the MDRD [13] = (186.3 * (serum creatinine)^–1.154^ * age^–0.203^ * 0.742 [for women]). Hypertension was defined as a blood pressure ≥140 mmHg and/or ≥90 mmHg, and/or use of antihypertensive medication. Diabetes type-2 was diagnosed with the criteria of the American Diabetes Association [15]. CHD was diagnosed by a history of myocardial infarction and revascularization, by angiographic greater than 50% stenosis of at least one major coronary artery.

### Outcome

Information on cause of death was drawn from hospital records during follow-up, from records of the nation-wide Identity Sharing System, or from first-degree relatives. Follow-up for mortality was complete via the Identity Sharing System which registers all deaths by using identity numbers for each citizen; causes were identified from hospital records or by phone from first-degree relatives. Information on nonfatal events at follow-up was obtained (in 70%) from hospital records at their return visits to the medical center, and the remainder by phone from close relatives of patients, Stroke was defined as affected impairment of brain function having resulted in an inability to move a limb, or inability to understand or formulate speech. Congestive heart failure (HF) was diagnosed when typical symptoms (e.g. breathlessness, ankle swelling, and fatigue) and signs (e.g. elevated jugular venous pressure, pulmonary crackles, and displaced apex beat) due to an abnormality of cardiac structure. Chronic obstructive pulmonary disease (COPD) designated patients who had no evidence of this at baseline but received this diagnosis in the follow-up period in our out-patient chest clinic, based on obstructive findings in pulmonary function testing and in whom bronchodilator drugs were initiated.

### Data analysis

Descriptive parameters were shown as mean (±standard deviation [SD]) or in percentages. Due to skewed distribution, geometric means were used for CRP values. Two-sided t-tests and Pearson’s chi-square tests were used to analyze the differences between means and proportions of variables within quartiles of eGFR and by pairwise comparisons with post hoc Tukey homogeneous subsets (HSD) tests. The development of multiple cardiopulmonary events in an individual was counted only as one subject, or in the case of death as death alone. Rather than defining fixed cutoffs for hyperfiltration for which unanimity is lacking, we formed sex-specific quartiles of eGFR defined by each equation. Cutoff points were 60–88, up to 99, up to 110, and >110 ml/min per 1.73 m^2^ for CKD-EPI, while these were <83.6/<82.6, up to 100/105, up to 132/141.9, >132/≥142 ml/min per 1.73 m^2^, respectively, for men/women in the MDRD formula. A multiple linear regression model, selected from findings of univariate correlation, was analyzed to seek best independent covariates of eGFR. Logistic regression analyses were used for combined endpoint of death and incident cardiovascular and pulmonary events (MI, HF, stroke and COPD) for eGFR quartiles formed by the two sets of cutoffs. Risk estimates (RR) and 95% confidence intervals (CI) were obtained in models that adjusted for sex, age and relevant confounders, expressed in terms of 1-SD increment. A value of p < 0.05 on the two-tail test was considered statistically significant. Statistical analyses were performed using SPSS-10 for Windows.

## Results

**Figure 1:**
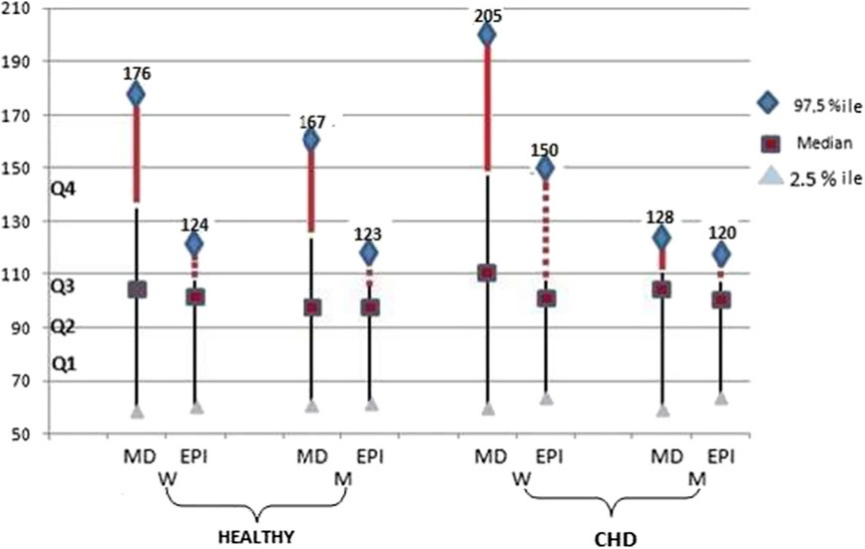
**Depicts graphically the median, 2.5 and 97.5 percentile GFR values estimated by the MDRD and CKD-EPI equations in healthy subjects and in those with CHD, free of chronic kidney disease, stratified by gender.** Noteworthy is that differences exist between the two equation-derived estimates only in quartiles 4 (depicted in thick lines) of healthy adults and in females, but not males with CHD, implicating that serum creatinine is little involved in an autoimmune complex in men during the development of CHD.

The study sample consisted of 403 women (age 55.9 ± 8.4) and 386 men (age 55.3 ± 8.0), age range 40 to 72 years. Median, 2.5 and 97.5 percentile eGFR values estimated by the MDRD and CKD-EPI equations in healthy subjects free of chronic kidney disease and those with (stable and acute) CHD are presented graphically in Figure 1, stratified by gender. CHD was diagnosed at baseline in 222 men and 98 women. Diabetes was identified at baseline in 213 subjects (27%). A total of 25 deaths and 75 instances of nonfatal MI, HF, stroke and COPD events occurred in 100 unique subjects during a follow-up of up to 3.0 (median 1.8) years. Additional instances of HF, nonfatal MI, stroke and COPD identified in patients who died or sustained an AMI were not included in analyses.

Table 1 shows prevalence of type-2 diabetes, clinical and biochemical data at baseline, stratified to presence of baseline CHD and eGFR quartiles. Among individuals free of CHD at baseline, the distribution of sex and diabetes was similar across the quartiles. The highest eGFR quartile differed from Q2 or Q3, the normal renal function group, in being younger, having significantly lower LDL-, HDL-, and total cholesterol, uric acid levels, and higher fasting triglycerides and tended to lower LVEF. In patients with prevalent CHD, significant difference across the quartiles was confined to the “hyperfiltrating” quartile displaying significantly younger age and lower uric acid levels.

**Table 1:** **Baseline characteristics of the study sample, by presence of baseline CHD and GFR quartiles estimated by CKD-EPI equation (n = 789)**

		Quartile 1	Quartile 2	Quartile 3	Quartile 4	
	Ren. fnct.	Mild dysfnct.	Normal	Normal	“Hyperfiltrator”	
	n	Mean ± SD	Mean ± SD	Mean ± SD	Mean ± SD	p-value
***No CHD*** (n = 469)						
Sex, n, M/F	164/305	41/76	42/77	40/76	41/76	1.00
eGFR,ml/min per 1.73 m^2^		74 ± 8.6	93.7 ± 4	104.2 ± 3.3	117.3 ± 6.8	
Creatinine, mg/dl		0.947 ± 0.14	.763 ± 0.14	.642 ± 0.14	.534 ± 0.11	
Age, yrs		59.7 ± 6.8	57.4 ± 7.4	54.6 ± 8	**47.8** ± 6	<0.001
BMI, kg/m^2^		28 ± 4	27.8 ± 3.1	27.2 ± 4	27.1 ± 3.8	0.20
Systolic BP, mmHg		129.4 ± 20	126 ± 19	125.6 ± 20	123.8 ± 19	0.16
Diastolic BP, mmHg		79.3 ± 10.4	77.7 ± 12	78.4 ± 10.4	75.9 ± 9.7	0.10
Total cholest, mg/dl	448	214 ± 49	213.5 ± 48	212 ± 47	**198** ± 43	0.042
LDL cholest, mg/dl	445	132 ± 39	130.5 ± 38	129 ± 39	**112.5** ± 32.4	<0.001
HDL cholest, mg/dl	448	53.1 ± 13.5	53.2 ± 13.4	**49.6** ± 14	**47.6** ± 16	0.007
F.triglycerides, mg/dl	448	154 ± 72	157 ± 82	162 ± 97	**195** ± 149	0.014
Fast glucose, mg/dl	467	110 ± 30	115 ± 52	117 ± 43	117 ± 34	0.51
HbA1c,%l	423	5.98 ± 0.8	5.95 ± 1.1	6.06 ± 1.1	5.91 ± 1.13	0.75
Uric acid, mg/dl	385	5.43 ± 1.1	5.2 ± 1.3	**4.97** ± 1.2	**4.79** ± 1.17	0.001
C-reactive protein,¶ mg/L	371	2.42 *x*2.74	2.64 *x*2.64	2.61 *x*2.61	2.38 *x*2.39	0.81
Current; past smokers,%		15.2; **29.8**	22.6; 19.3	27.6; 17.5	34.8; **33.3**	<0.001
LV ejection fraction,%	273	60.5 ± 7	60.1 ± 6	59.3 ± 5.7	*57.2* ± 11	0.058
Diabetes baseline, n,%		22; 18.8	23; 19.3	30; 25.9	26; 22.2	0.54
***CHD*** (n = 320)	Ren. fnct.	Mild dysfnct.	Normal	Normal	“Hyperfiltrator”	
Sex, n, M/F	222/98	54/27	49/26	58/25	61/20	0.53
eGFR,ml/min per 1.73 m^2^	320	74.3 ± 8	93 ± 3.6	103.6 ± 3.6	115.3 ± 6	
Creatinine, mg/dl		1.02 ± 0.15	0.81 ± 0.14.6	0.673 ± 0.14	0.589 ± 0.103	
Age, yrs	320	59.6 ± 7	58.7 ± 7.3	57.5 ± 7.7	**50.6** ± 6.6	<0.001
BMI, kg/m^2^		27.5 ± 3.9	28.3 ± 4	26.9 ± 3	27.6 ± 4.1	0.14
Systolic BP, mmHg		132.8 ± 18	134 ± 17	134 ± 16.5	132.3 ± 21	0.95
Diastolic BP, mmHg		81.3 ± 11	82.2 ± 9	80.6 ± 9.9	81 ± 9.5	0.78
Total cholest, mg/dl	315	191 ± 42	195 ± 42.5	187 ± 48	193 ± 39	0.73
LDL cholest, mg/dl	312	113 ± 32	118 ± 35	112.6 ± 36	116.9 ± 36	0.71
HDL cholest, mg/dl	315	43.7 ± 12	43.8 ± 11	42.3 ± 12	41 ± 9.6	0.36
F. triglycerides, mg/dl	315	168 ± 82	168 ± 86	164 ± 93	177 ± 100	0.85
Fast glucose, mg/dl		124 ± 58	124 ± 45	129 ± 52	129.6 ± 72	0.87
HbA1c,%	312	6.27 ± 1.24	6.28 ± 1.13	6.40 ± 1.50	6.61 ± 1.69	0.40
Uric acid, mg/dl	293	5.77 ± 1.4	5.47 ± 1.2	**5.21 ± 1.4**	**5.26 ± 1.15**	0.038
C-reactive protein,¶ mg/L	291	3.20 x3.2	3.35 *x*2.7	3.25 x3.37	4.24 x3.5	0.41
Current; past smokers,%		33.3; 45.7	44; 37.3	47; 37.3	55.6; 31.3	0.21
LV ejection fraction,%	243	51 ± 11.7	51.3 ± 11.4	51.8 ± 10	49.3 ± 11.6	0.63
Diabetes baseline, n,%		27; 33.3	27; 36	30; 36.1	28; 34.6	0.98

¶log-transformed values, SD range is obtained by dividing or multiplying with the given SD.

Numbers in boldface denote significant difference from Q2.

Number of subjects deceased or having incurred cardiopulmonary events at follow-up are provided in Table 2, by presence of CHD and quartiles of eGFR. MI, heart failure and cancer constituted the majority of causes of deaths. Of the 13 cases of new COPD identified in the follow-up (including one as cause of death) only 3 men and one woman were current smokers, whereas 9 men and women were never or former smokers.

**Table 2:** **Number of individuals free of or with coronary heart disease (CHD) at baseline who died or incurred cardiopulmonary events at follow-up, stratified to quartiles of eGFR (by CKD-EPI)**

	Total “healthy” sample			CHD at baseline		
	Total subjects	p = 0.37 Deaths	Nonfatal Events	Total subjects	p = 0.036 Deaths	Nonfatal Events
Quartile 1	117	1	4	81	3	14
Quartile 2	119	4	4	75		16
Quartile 3	116	6	3	83	1	19
Quartile 4	117	5	7	81	5	8
Grand total	469	16¶	18*	320	9¶	57‡

*As nonfatal events heart failure occurred in 13, chronic obstructive pulmonary disease (COPD) in 5 (all in quartiles 3 & 4) unique subjects

‡ Nonfatal myocardial infarction (MI) in 23, heart failure in 24, COPD in 8, stroke in 2 subjects.

¶ Death was due to MI in 6, heart failure in 5 patients, stroke and COPD in 2 patients each, lung cancer in 4, breast cancer in 2 patients, cardiomyopathy and leg amputation in 1 patient each. Cause remained obscure in 2 instances.

### Serum uric acid best covariate of eGFR

Five variables showing highest univariate correlations with eGFR were included in a multiple linear regression model to determine its independent covariates (Table 3). Using eGFR derived from both equations in highly significant regression models, serum uric acid levels proved a highly significant inverse covariate in the “healthy” group, independent of BMI and apart from age. LVEF was associated inversely with eGFR, significantly in the “healthy” group, tended weakly so in the CHD group.

**Table 3:** **Linear regression analysis for independent association with eGFR¶ using MDRD equation**

	Free of CHD, n = 221*			With CHD, n = 220*		
	ß coeff.	SE	p-value	ß coeff.	SE	p-value
Age, 8 years	**-12.5**	2.6	<0.001	**-8.7**	2.8	0.02
Uric acid, 1 mg/dl	**-6.4**	2.1	<0.001	**-3.4**	1.57	0.03
Diastolic b. pressure, 10 mmHg	-1.0	2.5	0.69	*3.5*	2.9	0.091
Body mass index, 4 kg/m^2^	-3.7	3.1	0.23	*-4.0*	2.3	0.093
LV ejection fraction, 10%	**-11.1**	3.3	0.001	*-3.9*	1.9	0.059
r^2^	18%, p < 0.001			7.5%, p = 0.001		
eGFR with CKD-EPI						
Age, 8 years	**-9.6**	1.0	<0.001	**-7.5**	1.0	<0.001
Uric acid, 1 mg/dl	**-3.0**	0.8	0.001	**-1.7**	0.8	0.035
Diastolic b. pressure, 10 mmHg	-0.03	1.0	0.97	**2.0**	1.0	0.047
Body mass index, 4 kg/m^2^	-1.1	1.2	0.34	-3.6	1.1	0.20
LV ejection fraction, 10%	**-4.4**	1.3	0.001	-1.4	0.9	0.12
r^2^	37%, p < 0.001			22%, p < 0.001		

*Limited by unavailable values for mainly left ventricular (LV) ejection fraction and, partly, serum uric acid.

CHD, coronary heart disease Numbers in boldface denote significantly associated values.

### Logistic regression for outcome

Table 4 shows predictors of combined outcome (death and nonfatal cardiopulmonary events) among the groups without and with CHD at baseline using the same regression model with eGFR derived from two equations. Among the “healthy” group, beyond age and former smoking, highest eGFR quartile with the MDRD equation significantly predicted the combined outcome at a RR of 4.00 (95%CI 1.34; 11.9) compared to the normal Q2, while sex, BMI and presence of hypertension did not reach significance. RR with the CKD-EPI was 2.94 (whereby p-trend =0.048), or attained a p = 0.006 when expressed using Q1 as referent (Table 4). Highest eGFR quartile significantly predicted outcome among patients with CHD at an RR 2.72 (95% CI 1.08; 6.81) yet only with the MDRD estimate, not with that of CKD-EPI.

**Table 4:** **Logistic regression models for combined death and nonfatal events, stratified by presence of CHD at baseline, comparing eGFR with both the MDRD and CKD-EPI equations**

	Free of CHD		CHD	
	RR	95% CI	RR	95% CI
*Combined events/Total at risk*	34/469		66/320	
eGFR with MDRD				
Sex, male	1.32	0.61; 2.85	1.16	0.60; 2.24
Age, 8 years	**1.53**	1.03; 2.32	**1.71**	1.24; 2.35
eGFR Quartile 1*, 69 ml/min/	0.78	0.21; 2.86	1.85	0.75; 4.53
eGFR Quartile 3*, 115 ml/min/	2.25	0.94; 6.88	*2.28*	0.96; 5.42
eGFR Quartile 4*, 163 ml/min/	**4.00**	1.34; 11.9	**2.72**	1.08; 6.81
Hypertension, yes/no	1.34	0.62; 2.89	0.83	0.46; 1.49
Body mass index, 4 kg/m^2^	1.06	0.71; 1.57	1.23	0.91; 1.64
Current vs never smoking	1.37	0.52; 3.57	1.06	0.45; 2.50
Former vs never smoking	**2.85**	1.08; 7.56	1.06	0.45; 2.49
eGFR with CKD-EPI				
Sex, male	1.31	0.61; 2.80	0.85	0.44; 1.62
Age, 8 years	**1.78**	1.13; 2.85	**1.73**	1.22; 2.48
eGFR Quartile 2*, 94 ml/min/1.73	2.07	0.64; 6.68	1.05	0.48; 2.31
eGFR Quartile 3*, 104 ml/min/1.73	*2.84*	0.87; 9.22	1.45	0.58; 3.11
eGFR Quartile 4*, 118 ml/min/1.73	**6.08Ť**	1.68; 22.0	1.36	0.55; 3.34
Hypertension, yes/no	1.30	0.61; 2.78	0.83	0.47; 1.48
Body mass index, 4 kg/m^2^	1.02	0.69; 1.53	1.20	0.90; 1.60
Current vs never smoking	1.30	0.51; 3.37	0.98	0.42; 2.29
Former vs never smoking	**2.74**	1.05; 7.18	0.98	0.42; 2.28

*Referent is expressed for quartile 2 in the upper, for quartile 1 (70 ml/min/) in the lower model.

Number of subjects in the group without and with CHD, respectively: 151/181 hypertensives; 164/144 current and 57/121 former smokers. CHD, coronary heart disease Ť p trend 0.048.

## Discussion

This study confirmed in middle-aged hospital applicants what has recently been proposed in a general population sample of Turkish adults, namely, that “hyperfiltrators” (usually persons with low serum creatinine) are actually at elevated cardiovascular risk. When stratified into quartiles of GFR estimated by the CKDI-EPI equation, “hyperfiltrators” in the “healthy” sample, were significantly younger, more frequently former smokers, had higher plasma atherogenic index (triglyceride/HDL-C ratio) and lower LDL-cholesterol, uric acid and LVEF, despite having similar BMI, compared to subjects with normal renal function. We are the first to determine prospectively that the highest eGFR quartile independently predicted the combined outcome of total death and cardiopulmonary events at roughly a 3-fold relative risk, irrespective of estimating GFR by the CKD-EPI or the MDRD equation. The risk profile among “hyperfiltrators” suggested an underlying autoimmune activation involving serum creatinine which rendered partial escape from immunoassay leading to misclassification of subjects having (seemingly paradoxically) elevated cardiovascular risk.

### Higher eGFR in females but “hyperfiltrators” comprise males as well

Individuals included in CKDI-EPI-derived eGFR Q2 or Q3 may be considered to represent normal renal function, displaying a range of 88–110 ml/min per 1.73 m^2^. Healthy females had eGFR similar to men while females with CHD had even somewhat higher eGFR than men, although in the general TARF sample [16] they had one-tenth lower values than men. This current finding is consistent with Turkish women being more susceptible to autoimmune activation than men, with resultant apparent “hyperfiltration” [12].

The current study demonstrates that, in a sample of subjects undergoing health checks, men as well are subject to Lp (a)-activated autoimmunity comprising serum creatinine. By showing similar distribution of the two GFR estimates across the quartiles in men with CHD, this study supported the conclusion by the TARF [9, 10] that, in contrast to females, serum creatinine is little involved in an autoimmune complex in men during the development of CHD, but is rather so in healthy men mainly regarding other outcomes such as heart failure, stroke and COPD.

### Rationale for including COPD as an outcome target

Although tobacco smoking is a major risk factor for COPD, recent evidence suggests that other risk factors are important as well, especially in developing countries [17]. One-third of patients with COPD are estimated to be never smokers but are thought to be exposed to biomass fumes [18–20], air pollutants, etc. Beyond this, enhanced pro-inflammatory state associated with obesity might result also in COPD among non-smokers, a tendency reported among Korean men [20]. On this postulate, we included COPD in this study as a potentially informative outcome and observed that all 5 cases of newly developing COPD in the “healthy” group belonged to baseline eGFR quartiles 3 and 4.

It is worth pointing out in this context that discontinuance of smoking was a significant predictor of combined outcome events in the multivariable regression model, suggesting inhibition of autoimmune processes by active smoking and being in line with the findings and interpretation regarding autoimmune type-2 diabetes developing in Norwegian men and women [21].

### Further distinctive risk profile of “hyperfiltrators”

That low serum creatinine was not due to low muscle mass associated with end-stage renal disease, or advanced heart failure, can be inferred from the younger age, similar BMI and LVEF of hyperfiltrators compared to participants in the remaining categories. Furthermore, a “reverse causality” is highly unlikely due to the prospective design of the study. Multiple linear analyses indicated that BMI was not independently related to hyperfiltration. Conversely, these patients were not undernourished, as evidenced by their being overweight in general. They were associated with atherogenic (high triglyceride/low HDL-cholesterol) dyslipidemia and exhibited, notably, lower concentrations of LDL- and total cholesterol than subjects with normal eGFR, similar to the large Swedish AMORIS study cohort [22] in which the overwhelming majority with a normal eGFR had 12% lower LDL- and total cholesterol values than those with mildly reduced eGFR. Yet when classified into quartiles, multivariable adjustment revealed in the highest eGFR quartile higher risk for all-cause mortality than the optimal risk quartile (>96 ml/min vs. 85–96 ml/min per 1.73 m^2^) and for incident MI than all the remaining eGFR quartiles.

Lower uric acid values among “hyperfiltrators” suggest the concomitant involvement in the immune complex of uric acid as well, for which some evidence was previously found in Turkish women [23, 24]. This consideration is in agreement with a large prospective study in Taiwan reporting that sex- and age-adjusted hazard ratios of uric acid levels for overall mortality were increased, also for uricemia lower than 5 mg/dl [25]. Our finding that “hyperfiltrators” were both younger and had lower serum uric acid are in line with recently reported observations among Turks that uric acid was independently and linearly associated with serum total phospholipids in men and women without MetS and that the concentration of phospholipids on HDL predicted the development of MetS [24], thus supporting a notion that –similar to creatinine– reduced uric acid level may be due to partial inability to assay, secondary to autoimmune activation. Puddu and Menotti [26] stressed that “serum uric acid might be considered for risk predictive purposes to be incorporated into software instruments aimed at indexing and/or following up individuals for primary preventive purposes.”

“Hyperfiltrators”, characterized by high serum triglycerides, suggested the presence of low circulating Lp(a) –by virtue of having damaged epitope that partly escapes immunoassay. Such an autoimmune “pattern” has been reported in the TARF study [12, 27]. Reduced levels of LDL-cholesterol have been reported to similarly precede the clinical onset of rheumatoid arthritis [28]. Epidemiological data showing paradoxical inverse association of lipid levels with cardiovascular disease risk in rheumatoid arthritis patients have been reviewed [29]. Furthermore, high serum triglycerides headed the determinants of a new cardiovascular event n 700 Swedish early rheumatoid arthritis patients [30].

Noteworthy is that the characteristics of atherogenic dyslipidemia and reduced LVEF of “hyperfiltrators” became diluted in the subset with baseline CHD, since CHD patients with normal renal function not only had diminished LVEF but also low total and LDL-cholesterol and a higher proportion of atherogenic dyslipidemia, eliminating the differences from the “hyperfiltrators”.

### Explanation of current results

Present findings support the hypothesized notion [12, 27] that autoimmune activation associated with enhanced low-grade inflammation is a common mechanistic pathway leading to diverse chronic diseases, foremost to diabetes, CHD and CKD. Serum creatinine, a tripeptide, may undergo oxidative damage to its epitope alike circulating a), be perceived as a foreign body, partly lose assayability and may aggregate to autoimmune components such as apoA-I [11] and/or adiponectin. Immune complexes with ß_2_-glycoprotein I-Lp (a) have been reported in patients with coronary artery disease [31]. The related individual is necessarily categorized into the lowest creatinine (or highest eGFR quantile), despite likely harboring a milieu of oxidative stress, HDL dysfunction and autoimmune activation associated with elevated cardiorenal-metabolic risk.

The reduced LVEF, along with our observation of HF as the most common consequence among “healthy” “hyperfiltrators”, suggests that such autoimmune activation frequently leads to HF the prevalence of which has been increasing in the community [32]. Cancer formed the second frequent death cause, being in line with a recent community-based case–control study that patients with heart failure have an increased risk of incident cancer [33], suggesting a common underlying mechanism.

### Implications

The magnitude of relative risk it conferred in this sample, and the substantial proportion in the population at large, necessitate the identification of potential “hyperfiltrators” in the community which has both methodological and clinical implications. One implication concerns the equation selected for estimating GFR, since the CKD-EPI [14], more suited to estimate creatinine-based GFR for the general population, tends to conceal “hyperfiltrators” by truncating creatinine values <0.7/<0.9 mg/dl. The MDRD estimate has the advantage of disclosing apparent “hyperfiltrators”, as can be inferred from analyzing eGFR quartiles in the current as well as the AMORIS study [22], commented above.

For clinically suspecting “hyperfiltrating” individuals, we may propose as a rough guide for 55-year old Turks an eGFR of >120/110 ml/min per 1.73 m^2^ (or serum creatinine <0.72 and <0.60 mg/dl) in men and women, respectively. The need for better understanding the mechanisms involved in renal hyperfiltration has been emphasized in order to develop new therapeutic strategies [1]. Much further research is warranted in this area in different ethnicities.

### Limitations and strength

The size of the study sample and the duration of the follow-up period are relatively limited which, however, did not preclude the emergence of significant results in the sample. Applicability of current findings, especially the extent of such in other ethnicities remains to be confirmed, but arguments may apply to similar findings among Swedes [22]. Given that underlying pathways examined here had already been published in representative samples of Turkish adults, we generated congruent results in patients suspected of heart disease undergoing health checks. Findings regarding COPD, obviously, may be considered only as preliminary and hypothesis generating. Strength of the study is its unique longitudinal design, as well as the stratified analyses of a sample susceptible to pro-inflammatory state, the availability of various clinical, lipid and inflammation biomarkers, including LVEF, thus supporting and confirming a novel hypothesis. The salient information provided by the concomitant evaluation of GFR estimates and their relative predictive value of selected outcomes is thus far unique.

## Conclusions

A sample of applicants of health check-up demonstrated that creatinine-based renal “hyperfiltrators prevailed in excess of one-third of the sample. They were characterized, not by a higher BMI, but by being younger and more frequently former smokers, having lower serum total and LDL-cholesterol, uric acid and LVEF, and a higher proportion of atherogenic dyslipidemia than persons with normal eGFR. Logistic regression models using two different creatinine-based eGFR at follow-up revealed significantly raised relative risk for combined death, cardiovascular and pulmonary events, above all HF, among the “hyperfiltrators” than in the subsets with normal eGFR, after adjustment for BMI and traditional cardiovascular risk factors. We conclude that the fundamental oxidative stress and autoimmune process simultaneously render partial escape of serum creatinine from full assay and impart excess risk of chronic cardiometabolic, renal and pulmonary morbidity and mortality. Clinicians need to be aware that unexpectedly low serum creatinine levels may represent a clue to the existence of autoimmune activation.
